# Reviewed article: Penha SC, Carioca AAF, Pinto MS, Adriano LS, Bastos MF, Herculano RPB, Kendall BC, Kerr LRF S. Food and Nutrition Surveillance System: A time series analysis of data coverage, ultra-processed food consumption and obesity among pregnant adults and adolescents, Brazil, 2008-2022. Epidemiol Serv Saude. 2025:34;e20240713

**DOI:** 10.1590/S2237-96222025v34e20240713.b

**Published:** 2025-11-21

**Authors:** Carla Cristina Enes

**Affiliations:** 1 Pontifícia Universidade Católica de Campinas, Campinas, SP, Brazil

## First round comments

Prezados autores,

Após análise pelos revisores, destacam-se os seguintes pontos para aprimoramento do manuscrito:

A seção de métodos necessita de maior detalhamento para garantir a reprodutibilidade do estudo, especialmente quanto à descrição das variáveis analisadas, procedimentos para cálculo de intervalos de confiança e utilização do SINASC. Foi sugerida a inclusão de dados mais específicos sobre o consumo de alimentos ultraprocessados, considerando grupos alimentares heterogêneos, e a padronização do número de casas decimais em todas as tabelas e seções de resultados. Além disso, a apresentação dos dados deve seguir a sequência do título, garantindo maior clareza ao leitor.

É necessário aprofundar a discussão com estudos similares realizados em outros países, permitindo uma análise comparativa mais rica. É necessário revisar o primeiro parágrafo da discussão para torná-lo mais claro e explicitar a relevância da Vigilância Alimentar e Nutricional, incluindo sua integração no sistema de saúde. Sugere-se ainda uma reflexão mais robusta sobre as limitações do estudo, considerando as restrições inerentes ao uso de dados secundários e potenciais vieses introduzidos.

O manuscrito se beneficiaria de ajustes gramaticais e ortográficos, uniformização do uso da sigla SISVAN e melhoria na fluidez do texto em trechos específicos. O resumo requer equilíbrio entre a descrição metodológica e os resultados, enquanto alguns trechos do texto, como as associações entre consumo de alimentos ultraprocessados e desfechos de saúde, necessitam de maior clareza e suporte com referências adicionais. 

Recommendation: Minor revision

